# Autoimmune Gastritis Diagnosis: Encompassing Pessimism, Realism, and Wish for the Future. Comment on Vienneau et al. Autoimmune Metaplastic Atrophic Gastritis Reporting: Are Pathologists and Endoscopists on the Same Page? *Diagnostics* 2025, *15*, 2906

**DOI:** 10.3390/diagnostics16071073

**Published:** 2026-04-02

**Authors:** Massimo Rugge

**Affiliations:** General Anatomic Pathology and Cytopathology Unit, Department of Medicine DIMED, University of Padova, Via Aristide Gabelli, 61, 35121 Padova, Italy; massimo.rugge@unipd.it

I have read with great interest the manuscript by N. Vienneau and coauthors on autoimmune gastritis (AIG) [1]. From their pathology database, the authors identified sixty-three reports that included “final diagnoses or comments mentioning the possibility of AMAG (i.e., Autoimmune Metaplastic Atrophic Gastritis) or showing well-differentiated neuroendocrine tumor (NET).”

After a thorough reevaluation of the cases, the authors confirmed the original diagnostic proposal and provided a detailed description of the well-established clinical–pathological profile of atrophic autoimmune gastritis [2,3,4,5].

In addition to acknowledging the rigorous nature of the study, I would like to focus on the manuscript’s title and content. Both effectively reflect the manuscript’s conclusions: “Enhanced adherence to biopsy guidelines, standardized pathology reporting, and consistent surveillance, especially for patients with AMAG without NET, are essential for improving diagnosis and outcomes [1].”

I would like to briefly comment on the cultural and clinical contexts that shape the authors’ conclusions.

- “Enhanced adherence to biopsy guidelines”: Reducing costs in diagnostic procedures, including Esophagogastroduodenoscopy (EGD), has become a priority in both public and private clinical practice. Minimizing biopsy samples has mainly been warranted by the need to “protect” overwhelmed pathologists! Building on that assumption, endoscopists are reluctant to adhere to at least three well-established recommendations: (i) include in the submission of the biopsy specimens the indication(s) to the (EGD) procedure; (ii) perform gastric biopsy sampling according to internationally recognized, validated protocols; and (iii) submit gastric oxyntic and antral biopsy specimens in separate, topographically identified vials [6,7,8,9,10,11]. It should be considered, however, that the clinical indication for EGD is most often generated by specialists or general practitioners, who are detached from endoscopy rooms and—even more so—from pathologists and their microscopes. In addition to administrative details such as age and sex, requests for histology examinations often lack clinical indications, details of comorbidities, reports of prior endoscopies or biopsies, and relevant laboratory findings. As a consequence, both endoscopists and, even more so, pathologists are blinded from the original clinical question and restrict their diagnostic commitment to answer three chief questions: (i) Are there lesions at risk of bleeding? (ii) Is there *H. pylori* infection? (iii) Can we exclude malignancies? Suggesting the etiological hypothesis of AIG requires more than that.
- “Standardized pathology reporting”: The authors reference two international guidelines that specifically address histology reporting [8,12,13]. However, these guidelines cover only a small part of the extensive literature on the subject. While adhering to guidelines can promote compliance with standard reporting, this remains an uncommon practice. Additionally, in rare diseases, pathologists often prefer descriptive diagnoses that omit comments on the gastritis phenotype and its cause. This ‘minimalistic’ descriptive approach, developed by clinically blinded pathologists, prevents criticism of diagnostic redundancy and excludes epicritic analysis of the histological lesion pattern. However, are these the expectations held by both clinicians and patients?
- “Consistent surveillance”: Current clinical practice is based on national and international guidelines. Recent evidence suggests that, in the absence of preexisting *H. pylori*-atrophic damage, autoimmune gastritis does not significantly increase the risk of epithelial malignancy. Many previous studies that indicated a cancer risk associated with pernicious anemia often overlooked the presence of *H. pylori* comorbidity. Future research should focus on this condition, and more definitive recommendations for endoscopic and biopsy follow-up should be made to address secondary cancer prevention in gastric comorbidities [14,15,16].

The disappointing outcome of these overlapping conditions is that (i) AIG is often clinically unrecognized; (ii) the presumed low incidence of AIG should be supported by more robust epidemiological evidence based on innovative diagnostic tools; (iii) there is no consistent data on AIG epidemiological incidence and temporal trends; (iv) the association between AIG and the risk of gastric cancer remains unclear; (v) the follow-up schedule for AIG should primarily focus on the secondary prevention of neuroendocrine tumors (NETs).

In conclusion, the management of AIG patients in clinical practice reveals a significant lack of interdisciplinary communication that extends beyond endoscopists and pathologists. Highly qualified specialists, including gastroenterologists, general practitioners, endoscopists, pathologists, and surgeons, tend to focus mainly on their areas of expertise, often struggling to provide a collaborative, patient-centered clinical approach. Could Artificial Intelligence-driven clinical networking help close this gap [17]?

## Data Availability

No new data were created or analyzed in this study. Data sharing is not applicable to this article.

## References

[1] Vienneau N., Lee H., Zhang X., Park E., Cleary M., Zhou J., Tarar S., Liu M., Tadros M. (2025). Autoimmune Metaplastic Atrophic Gastritis Reporting: Are Pathologists and Endoscopists on the Same Page?. Diagnostics.

[2] Rugge M., Bricca L., Guzzinati S., Sacchi D., Pizzi M., Savarino E., Farinati F., Zorzi M., Fassan M., Dei Tos A.P. (2023). Autoimmune gastritis: Long-term natural history in naïve Helicobacter pylori-negative patients. Gut.

[3] Rugge M., Savarino E., Sbaraglia M., Bricca L., Malfertheiner P. (2021). Gastritis: The clinico-pathological spectrum. Dig. Liver Dis..

[4] Rugge M., Fassan M., Pizzi M., Zorzetto V., Maddalo G., Realdon S., De Bernard M., Betterle C., Cappellesso R., Pennelli G. (2012). Autoimmune gastritis: Histology phenotype and OLGA staging. Aliment. Pharmacol. Ther..

[5] Lenti M.V., Rugge M., Lahner E., Miceli E., Toh B.H., Genta R.M., De Block C., Hershko C., Di Sabatino A. (2020). Autoimmune gastritis. Nat. Rev. Dis. Primers.

[6] Nieuwenburg S.A.V., Waddingham W.W., Graham D., Rodriguez-Justo M., Biermann K., Kuipers E.J., Banks M., Jansen M., Spaander M.C.W. (2019). Accuracy of endoscopic staging and targeted biopsies for routine gastric intestinal metaplasia and gastric atrophy evaluation study protocol of a prospective, cohort study: The estimate study. BMJ Open.

[7] Banks M., Graham D., Jansen M., Gotoda T., Coda S., di Pietro M., Uedo N., Bhandari P., Pritchard D.M., Kuipers E.J. (2019). British Society of Gastroenterology guidelines on the diagnosis and management of patients at risk of gastric adenocarcinoma. Gut.

[8] Malfertheiner P., Megraud F., Rokkas T., Gisbert J.P., Liou J.M., Schulz C., Gasbarrini A., Hunt R.H., Leja M., O’Morain C. (2022). Management of *Helicobacter pylori* infection: The Maastricht VI/Florence consensus report. Gut.

[9] Bornschein J., Tran-Nguyen T., Fernandez-Esparrach G., Ash S., Balaguer F., Bird-Lieberman E.L., Córdova H., Dzerve Z., Fassan M., Leja M. (2021). Biopsy Sampling in Upper Gastrointestinal Endoscopy: A Survey from 10 Tertiary Referral Centres Across Europe. Dig. Dis..

[10] Latorre G., Vargas J.I., Shah S.C., Ivanovic-Zuvic D., Achurra P., Fritzsche M., Leung J.S., Ramos B., Jensen E., Uribe J. (2024). Implementation of the updated Sydney system biopsy protocol improves the diagnostic yield of gastric preneoplastic conditions: Results from a real-world study. Gastroenterol. Hepatol..

[11] El-Zimaity H. (2008). Gastritis and gastric atrophy. Curr. Opin. Gastroenterol..

[12] Rugge M., Pennelli G., Pilozzi E., Fassan M., Ingravallo G., Russo V.M., Di Mario F., Gruppo Italiano Patologi Apparato Digerente (GIPAD), Società Italiana di Anatomia Patologica e Citopatologia Diagnostica/International Academy of Pathology, Italian Division (SIAPEC/IAP) (2011). Gastritis: The histology report. Dig. Liver Dis..

[13] Rugge M., Genta R.M., Malfertheiner P., Dinis-Ribeiro M., El-Serag H., Graham D.Y., Kuipers E.J., Leung W.K., Park J.Y., Rokkas T. (2024). RE.GA.IN.: The Real-world Gastritis Initiative-updating the updates. Gut.

[14] Vannella L., Lahner E., Osborn J., Annibale B. (2013). Systematic review: Gastric cancer incidence in pernicious anaemia. Aliment. Pharmacol. Ther..

[15] Rugge M., Fassan M., Pizzi M., Graham D.Y. (2013). Letter: Gastric cancer and pernicious anaemia—Often Helicobacter pylori in disguise. Aliment. Pharmacol. Ther..

[16] Murphy J.D., Gadalla S.M., Anderson L.A., Rabkin C.S., Cardwell C.R., Song M., Camargo M.C. (2024). Autoimmune conditions and gastric cancer risk in a population-based study in the United Kingdom. Br. J. Cancer.

[17] Rugge M., Fraschini M., Orvieto E., Didaci L., Sandona’ L., D’Amuri A., Saba L., Faa G. (2025). Patient safety in AI-powered diagnostic pathology. J. Clin. Pathol..

